# Trends of incidence and prognosis of gastric neuroendocrine neoplasms: a study based on SEER and our multicenter research

**DOI:** 10.1007/s10120-020-01046-8

**Published:** 2020-02-05

**Authors:** Ping Hu, Jian’an Bai, Min Liu, Jingwen Xue, Tiaotiao Chen, Rui Li, Xiaoling Kuai, Haijian Zhao, Xiaolin Li, Ye Tian, Wei Sun, Yujia Xiong, Qiyun Tang

**Affiliations:** 1 Department of Gerontology, The First Affiliated Hospital of Nanjing Medical University, 300 Guangzhou Rd, Nanjing, 210029 Jiangsu Province China; 2 The First Affiliated Hospital of Soochow University, Suzhou, China; 3 Affiliated Hospital of Nantong University, Nantong, China; 4 The Second People’s Hospital of Huai’an, Huai’an, China; 5 Huai’an First People’s Hospital, Huai’an, China

**Keywords:** Gastric neuroendocrine neoplasms, Incidence, Nomogram, Overall survival

## Abstract

**Background:**

To investigate the recent epidemiological trends of gastric neuroendocrine neoplasms (GNENs) and establish a new tool to estimate the prognosis of gastric neuroendocrine carcinoma (GNEC) and gastric neuroendocrine tumor (GNET).

**Methods:**

Nomograms were established based on a retrospective study on patients diagnosed with GNENs from 1975 to 2016 in Surveillance, Epidemiology and End Results database. External validation was performed among 246 GNENs patients in Jiangsu province to verify the discrimination and calibration of the nomograms.

**Results:**

The age-adjusted incidence of GNENs has increased from 0.309 to 6.149 per 1,000,000 persons in the past 4 decades. Multivariate analysis indicated independent prognostic factors for both GNEC and GNET including age, distant metastasis and surgical intervention (*P *< 0.05). In addition, T, N staging and grade were significantly associated with survival of GNEC, while size was a predictor for GNET (*P *< 0.05). The C-indexes of the nomograms were 0.840 for GNEC and 0.718 for GNET, which were higher than those of the 8th AJCC staging system (0.773 and 0.599). Excellent discrimination was observed in the validation cohorts (C-index of nomogram vs AJCC staging for GNEC: 0.743 vs 0.714; GNET: 0.945 vs 0.927). Survival rates predicted by nomograms were close to the actual survival rates in the calibration plots in both training and validation sets.

**Conclusions:**

The incidence of the GNENs is increasing steadily in the past 40 years. We established more excellent nomograms to predict the prognosis of GNENs than traditional staging system, helping clinicians to make tailored decisions.

**Electronic supplementary material:**

The online version of this article (10.1007/s10120-020-01046-8) contains supplementary material, which is available to authorized users.

## Introduction

Neuroendocrine neoplasms (NENs) is a group of highly heterogeneous tumors originating from peptidergic neuron and neuroendocrine cells. The incidence of NENs has increased to 6.98/100,000 [1] in 2012 according to the data of Surveillance, Epidemiology and End Results (SEER) database. The increase could be observed among NENs at all sites, especially in gastric NENs (GNENs) with nearly 15-fold in the past 40 years [1], reaching up to 4.85/1,000,000 in 2014 [2]. According to WHO classification of 2010, NENs have been divided into well-differentiated neuroendocrine tumor (NET) and poorly differentiated neuroendocrine carcinoma (NEC) [3]. The outcomes of GNENs varied significantly between gastric NEC (GNEC) and gastric NET (GNET). And the prognosis of GNEC remained unsatisfying, which was significantly poorer than that of gastric adenocarcinomas (GAC) [4, 5]. Compared with other gastrointestinal NENs, patients with GNENs also presented with much lower median survival rates [1, 6, 7]. However, there are no effective models or markers to predict the prognosis of patients with GNENs.

TNM staging proposed by American Joint Committee on Cancer (AJCC) is now one of the most important prognostic factors for gastroenteropancreatic NENs (GEP-NENs). Previous reports indicated that AJCC staging played an important role in predicting the survival rates of GEP-NENs [8, 9]. However, many other factors, such as age, treatment or grade, which were not involved in AJCC staging system might affect the outcomes of GEP-NENs as well [10–12]. Thus, we need a novel model or system which could combine all the effective clinicopathological features to provide more accurate prediction for patients with GEP-NENs.

Nomograms, a graphical calculation or algorithm with continuous scales to calculate the probability of a particular outcome, had shown a more effective predicted ability than traditional staging systems in many cancers, including GEP-NENs [13, 14]. However, previous nomograms were based on analysis of cohorts which mix NEC and NET together. As we all know, NEC presented more aggressive behavior with poorer prognosis than NET [15, 16].

In the present study, we tried to explore epidemiological characteristics of GNENs based on a retrospective study from SEER database. Then, we constructed two novel nomograms for GNEC and GNET to help clinicians to predict the survival more precisely. At last, we collected clinical data of patients with GNENs from eight hospitals in Jiangsu Province, China, and validated the effectiveness of the nomograms.

## Materials and methods

### Population

#### Cohorts to estimate trends of incidence of GNENs

Patients with GNENs were collected from SEER database which was submitted on November 2018. The primary site code (C16.0–C16.9, stomach) and the following International Classification of Diseases for Oncology, Third Edition (ICD-O-3) histology codes were used to identify cases with GNENs: 8013 (Large cell neuroendocrine carcinoma), 8246 (Neuroendocrine carcinoma), 8244 (Mixed adenoneuroendocrine carcinoma, MANEC), 8240 (Carcinoid tumor), 8249 (Atypical carcinoid tumor).

There are three SEER registry systems named SEER 9, SEER 13 and SEER 18, which cover approximately 9.4%, 13.4% and 27.8% of all the American population, respectively. To maximize the representativeness of our study, we calculated the incidences of GNENs in 1973–1991 with SEER 9, in 1992–1999 with SEER 13 and in 2000–2016 with SEER 18 databases.

#### Cohorts to analyze the survival trends of GNENs

Demographic or clinical information including age, gender, race, tumor site, tumor size, grade, TNM staging and treatment were extracted from the SEER database. Collaborative Stage Data Collection System was used as a supplement for the missing values in SEER database. TNM staging was redetermined according to the criterion of the 8th AJCC guidelines. Notably, different from the 2010 WHO grading nomenclature, the SEER database classifies tumors into grade I (well differentiated), grade II (moderately differentiated), grade III (poorly differentiated) and grade IV (undifferentiated/anaplastic) according to histological differentiation. In our study, grade III and grade IV were combined into one category and analyzed together. Patients who underwent photodynamic therapy, electrocautery, cryosurgery, laser excision, polypectomy, excisional biopsy were described as local resection. Those with endoscopic mucosal resection (EMR) or endoscopic submucosal dissection (ESD) were also included in the local resection group. Patients who underwent partial or total gastrectomy with lymphadenectomy were described as radical resection of the tumor.

**Fig. 1:**
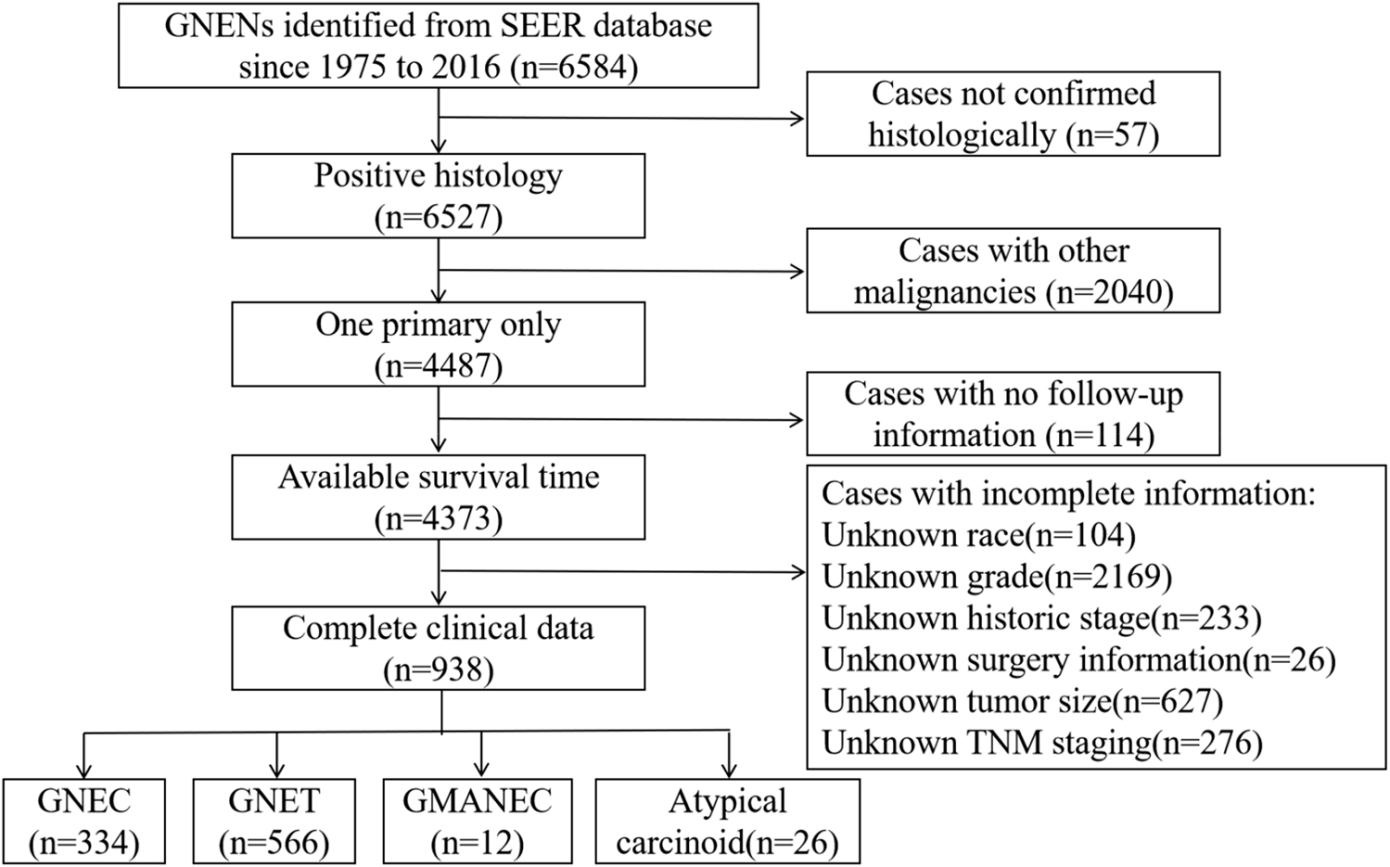
Schematic overview for patient identification

Among 6584 patients with GNENs identified from SEER database, only those confirmed by histopathology were included in the survival cohorts. Other exclusion criteria included: (1) cases with a history of other malignancies; (2) cases without follow-up information; (3) cases without complete clinical data mentioned above. Finally, a total of 334 cases with GNEC and 566 cases with GNET without missing values were assigned as training sets (Fig. 1).

In addition, another 139 GNEC patients and 107 GNET patients from eight tertiary hospitals in Jiangsu province were enrolled in our study as validation sets. All of these patients were diagnosed by two different pathologists according to the WHO classification of 2010. In order to ensure a sufficient follow-up time, only those diagnosed between January 2010 and December 2017 were eligible for our study. The other exclusion criteria mentioned above for training sets applied equally to the validation sets. The deadline of the follow-up was August 31, 2019. Patients who were alive at the last follow-up date were treated as censored observations. Survival time was defined as the duration from the diagnosis to death, last contact or August 31, 2019.

### Statistical analysis

Age-adjusted incidences standardized according to the 2000 US standard population were calculated with SEER * Stat software, version 8.2.5 (Surveillance Research Program, National Cancer Institute). Annual percentage changes (APCs) and log-linear models were used to assess the variation of incidence of GNENs with Joinpoint Regression Program version 4.7 (Surveillance Research Program, National Cancer Institute).

Survival analysis was performed with SPSS, version 25.0. Kaplan–Meier survival curves were constructed for each variable and were compared with log-rank test. The variables with a *P* < 0.1 from the univariate analysis were included in the Cox proportional hazards regression model to determine the risk factors associated with the prognosis of GNENs.

Then, nomograms were established based on the independent prognostic factors selected by the multivariate analysis from training sets. Discrimination of the nomograms was evaluated by Harrell’s concordance index (C-index). Moreover, the area under the receiver operating characteristic (ROC) curve (AUC) was also used to assess the performance of the prognostic models. The predicted survival rates were compared with the actual survival rates determined using a Kaplan–Meier analysis, and calibrations were generated. Bootstraps with 300 reiterations were used for these activities. The total points of the patients in the validation sets were calculated according to the corresponding nomograms. Then the total points were viewed as a new factor in the COX regression model, and the C-index, AUC and calibration curve were derived from the regression analysis in the external validation cohorts. The analysis was performed with R software, version 3.6.0 (https://www.r-project.org) via rms and survival package. *P* < 0.05 was considered as statistically significant.

This retrospective study was approved by the ethics committee of the First Affiliated Hospital of Nanjing Medical University.

## Results

### Incidence trends of GNENs

**Fig. 2:**
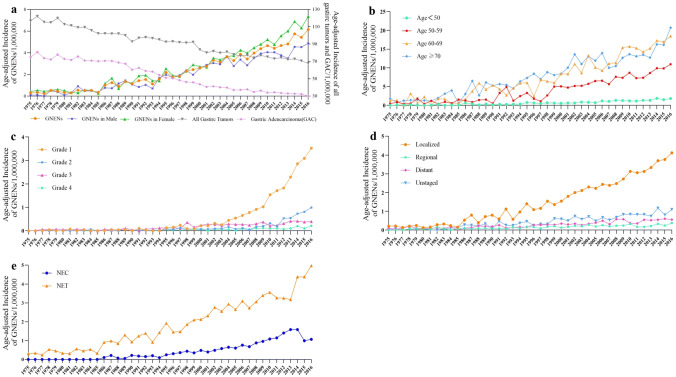
Annually age-adjusted incidence of GNENs. Total age-adjusted incidence of GNENs and by gender (**a**), by age (**b**), by grade (**c**), by stage (**d**), by histological types (**e**)

The age-adjusted incidence of GNENs increased steadily from 0.309/1,000,000 in 1975 to 6.149/1,000,000 in 2016 (Fig. 2a). A slightly higher incidence was observed in female than male during the same period. However, comparing with GNENs, the incidence of all the gastric tumors and GAC decreased in the past 40 years (Fig. 2a).

This phenomenon occurred among all the age groups, and the most dramatic rise was noted in patients older than 60 years old, nearly 12- and 14-fold in those 60–69 years and > 70 years, respectively (Fig. 2b). The same growth trends could be observed across all grades and stages, especially in grade I and localized tumors, reaching up to 3.525 and 4.118 per 1,000,000 persons, respectively (Fig. 2c, d). Compared with GNEC, the incidence of GNET increased more rapidly with over 16-fold rise to 4.978/1,000,000 (Fig. 2e).

In the period from 1975 to 2002, the average APC of the age-adjusted incidence of all GNENs was 8.6% (95% CI: 7.4–9.8%), while in the period from 2002 to 2016, the APC was 4.7% (95% CI = 3.9–5.5%, *P *< 0.05, Supplementary Figure 1).

### Survival trends and factors associated with the prognosis with GNENs

The demographic or clinical characteristics of patients with GNENs in training and validation sets was shown in Supplementary Table.

In SEER database, the median diagnosed age of GNEC was 63 years old (from 21 to 89), which was significantly older than the GNET patients (59 years old, from 17 to 85, *P *< 0.001).

In the primary GNEC cohort, the median survival time was 71 months and the 3-year, 5-year overall survival rate were 59.4%, 52.2%, respectively. The prognosis of patients with different gender, age, grade, size, tumor site, T staging, N staging, M staging, operation methods varied significantly in GNEC group with univariate analysis (Supplementary Figure 2A–J, *P *< 0.001). The 3-year OS of GNEC patients at AJCC I, II, III, IV staging were 91.4%, 86.9%, 42.5%, 18.7%, respectively. However, multivariate analysis indicated that only age, grade, T staging, N staging, M staging, operation methods were independent prognostic factors for GNEC (Table 1, *P *< 0.05).

**Table 1:** Multivariate analysis of the clinicopathological features of GNEC in SEER database

		*P*	HR	95% CI	
				Lower	Upper
Age	< 50		Reference		
	50-69	0.022	1.775	1.087	2.900
	≥ 70	< 0.001	2.803	1.717	4.577
Grade	G1		Reference		
	G2	0.038	2.010	1.039	3.888
	G3/4	< 0.001	4.333	2.506	7.429
T staging	T1/2		Reference		
	T3/4	0.035	1.509	1.030	2.210
N staging	N0		Reference		
	N1	< 0.001	2.154	1.503	3.088
M staging	M0		Reference		
	M1	< 0.001	2.373	1.657	3.399
Surgery	No surgery		Reference		
	Local	0.042	0.427	0.188	0.970
	Radical	< 0.001	0.359	0.240	0.537

*GNEC* gastric neuroendocrine carcinoma, *SEER* Surveillance, Epidemiology and End Results

As for the GNET group, the median OS was over 150 months, which was significantly longer than that of GNEC patients (71 months, *P *= 0.006). Meanwhile, the 3-year, 5-year overall survival rate of GNET were also better than that of GNEC and were 90.2%, 81.1%, respectively. Three-year OS were 92.6%, 89.8%, 93.5%, 40.9% for GNET patients at AJCC I, II, III, IV staging, respectively. Gender, age, tumor size, grade, N staging, M staging, surgery also affected the prognosis of GNET (Supplementary Figure 3A–J). However, only age, tumor size, M staging, surgery were independent prognostic factors of GNET (Table 2, *P *< 0.05). Different from GNEC, no significant differences were observed with respect to grade (*P *= 0.366), T staging (*P *= 0.999) or N staging (*P *= 0.376) in the multivariate analysis.

**Table 2:** Multivariate analysis of the clinicopathological features of GNET in SEER database

		*P*	HR	95% CI	
				Lower	Upper
Age	< 60		Reference		
	≥ 60	< 0.001	3.351	2.013	5.579
Size	≤ 2 cm		Reference		
	> 2 cm	< 0.001	2.679	1.545	4.646
M staging	M0		Reference		
	M1	< 0.001	5.465	2.595	11.512
Surgery	No surgery		Reference		
	Local	0.049	0.484	0.264	0.888
	Radical	0.019	0.544	0.297	0.998

*GNET* gastric neuroendocrine tumor, *SEER* Surveillance, Epidemiology and End Results

### Nomograms construction

**Fig. 3:**
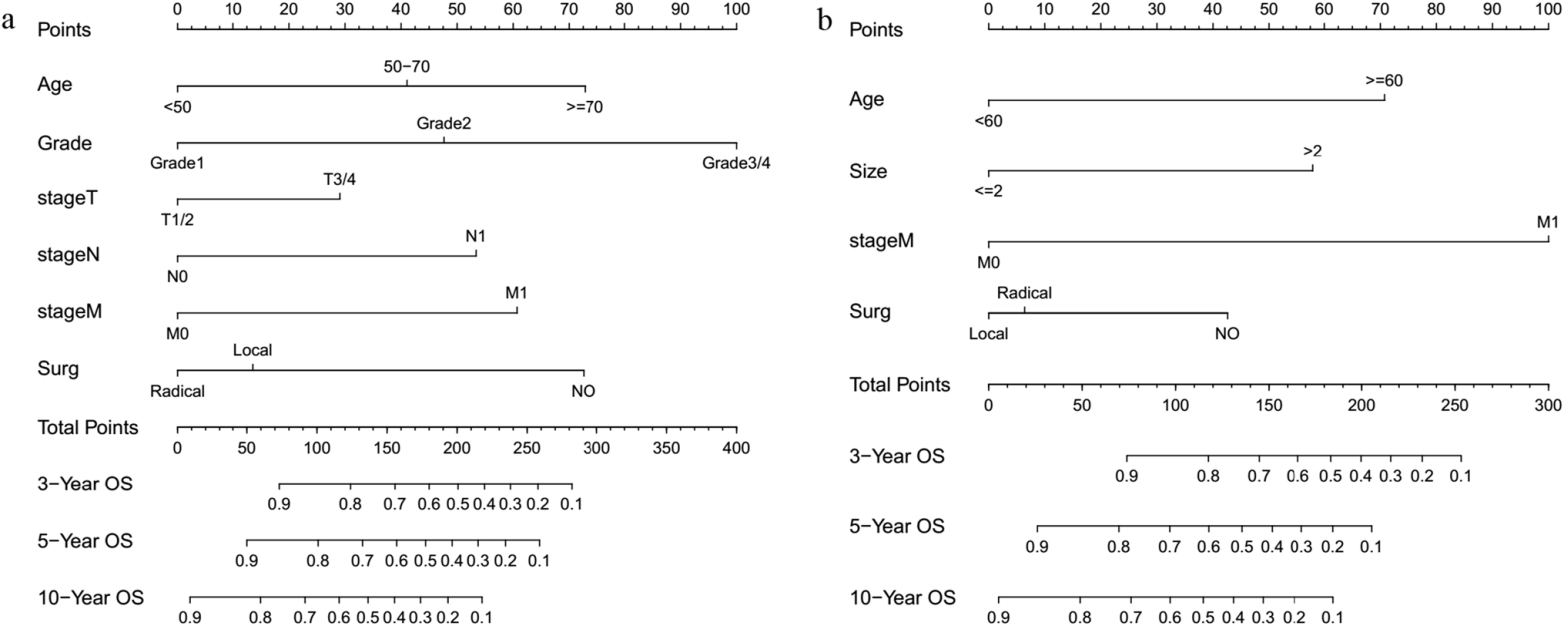
Nomograms to predict the 3-, 5-, 10-year overall survival of patients with GNEC (**a**) and GNET (**b**). Score for each independent prognostic factor was summed up. Then, the overall survival rate was estimated by the total points on the bottom scales for each individual

Nomograms were established based on the selected parameters via COX regression model to predict the long-term survival for patients with GNEC (Fig. 3a) and GNET (Fig. 3b). The C-indexes of the nomograms for OS in both GNEC and GNET training sets were superior to those of the 8th AJCC staging system [0.840 (95% CI = 0.811–0.869) vs 0.773 (95% CI = 0.740–0.806), 0.718 (95% CI = 0.653–0.782) vs 0.599 (95% CI = 0.530–0.668)].

**Fig. 4:**
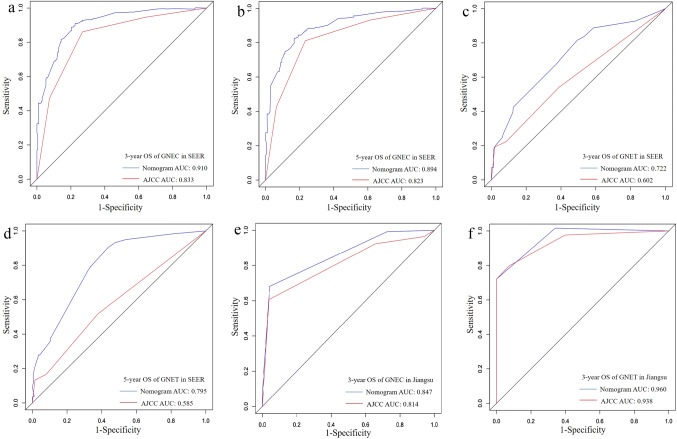
Comparison of the AUCs of the nomograms and the 8th AJCC TNM staging system. The areas under the curves of the nomograms to predict 3- and 5-year overall survival of GNEC (**a**, **b**) and GNET (**c**, **d**) in the training sets were larger than those of the 8th AJCC staging. Similar superiority of the nomograms also lied in predicting the OS at 3 year after the diagnosis of GNEC (**e**) or GNET (**f**) in Jiangsu validation sets

**Fig. 5:**
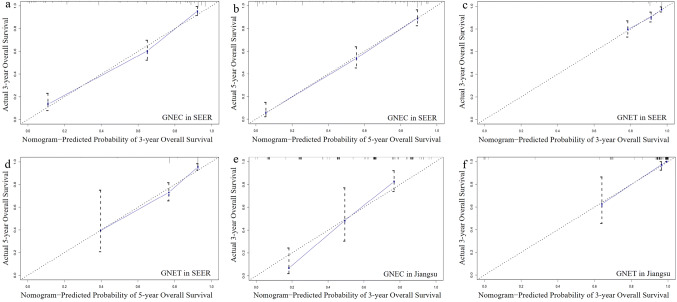
The calibration plots comparing the similarity between the nomogram-predicted survival rates (represented by *x*-axis) and the actual survival rates (represented by *y*-axis). **a** Three-year survival of GNEC in SEER database; **b** 5-year survival of GNEC in SEER database; **c** 3-year survival of GNET in SEER database; **d** 5-year survival of GNET in SEER database; **e** 3-year survival of GNEC in Jiangsu validation set; **f** 3-year survival of GNET in Jiangsu validation set

In the primary GNEC cohort, the AUCs of the nomogram for predicting the 3- and 5-year OS were 0.910 and 0.894, respectively, while the AUCs of the traditional AJCC staging system were 0.833 for 3-year OS and 0.823 for 5-year OS (Fig. 4a, b). In the GNET cohort, the AUCs of the nomogram were also larger than that of the traditional AJCC staging system for both 3-year OS (0.722 vs 0.602, Fig. 4c) and 5-year OS (0.795 vs 0.585) (Fig. 4d).

As shown in Fig. 5a–d, calibration plots were generated to validate the similarities between the survival prediction by the nomograms and the actual observation. In both the GNEC and GNET cohorts, we achieved an optimal agreement between the 3- and 5-year survival rates predicted by nomograms and the actual survival rates.

### Nomograms validation

In the external validation sets, we included 246 patients with GNENs from 8 tertiary hospitals in Jiangsu province, including 139 cases with GNEC and 107 with GNET. The median diagnosed age for GNEC and GNET were 66 years old (ranged from 42 to 82) and 55 years old (ranged from 17 to 89), significantly older than those in SEER database in GNEC group (*P *= 0.022) but younger in GNET patients (*P *= 0.049).

Among the 139 GNEC patients from Jiangsu, the median follow-up duration was 27 months (ranged from 2 to 89) and the median OS was approximately 3.4 years. The 3-year overall survival rate was 53.5% and continue to decrease to 47.1% at 60 months. With respect to the 107 cases with GNET, the median follow-up time was 40 months (ranged from 6 to 107) with a significantly better prognosis than GNEC (*P* < 0.05). The median OS was more than 8.9 years with a 3-year overall survival rate up to 89.6%.

As for GNEC patients, the C-index of the nomogram in the validation set was 0.743, which was higher than the AJCC staging system (0.714). In addition, the AUCs further confirmed the superiority of our nomogram to predict the 3-year survival (nomogram vs AJCC = 0.847 vs 0.814, Fig. 4e). With respect to GNET patients, both the C-index (nomogram vs AJCC = 0.945 vs 0.927) and AUC (nomogram *vs* AJCC = 0.960 vs 0.938, Fig. 4f) of the nomogram were higher than that of AJCC staging system. The calibration plots suggested that the predicted 3-year survival rate were consistent with the actual survival rate within an acceptable margin of error both in GNEC and GNET patients (Fig. 5e–f).

## Discussion

GNENs were an orphan disease and accounted for 6.9% of all the GEP-NENs, representing 0.3–1.8% of all gastric malignancies [17–19]. Based on the up-to-date SEER database, our study revealed that the incidence of GNENs has increased gradually in the past 4 decades, consisting with the previous study [1, 2, 17]. Moreover, it was rapidly increased among those with localized and grade I tumors. The possible reasons for a higher detection rate of tumors in early stage may attribute to development of endoscopic surveillance, more widespread biopsy of ‘simple’ polyps as well as improvement of immunohistochemistry techniques. What we should mention is that the actual incidence of GNENs may be underestimated because of the atypical symptoms and even symptomless.

However, GNENs remain poorly understood due to the rarity and high heterogeneity of the tumors. Effective risk stratification instruments are needed to make clinical guides. The most widely used AJCC staging has been questioned in prognosis prediction of GNEC patients [8]. Moreover, additional clinicopathological characteristics including age, Ki-67 index and therapeutic options have also been proven to be important predictors of GNENs [20–22]. We also found that an older patient had a significant higher risk to die from GNENs than the younger ones. A 50–60% reduction in the risk of death was observed in patients who removed the tumor than those who did not according to our study. However, we could not assess the importance of these factors by TNM staging system.

In our study, we established two nomograms to predict the prognosis of GNENs. We collected six independent prognostic factors for GNEC and four factors for GNET in our nomograms according to the multivariate analysis results, including age, distant metastasis, surgical modalities and so on. Both the C-index and the ROC curves of our nomograms were better than that of the eighth AJCC staging system, and the predicted 3- and 5-year OS were similar with the actual survival rates in the calibrations, indicating the established nomograms may help us predict 3-year and 5-year OS of GNENs more precisely.

Moreover, we performed a multivariate analysis for GNEC or GNET separately and found some discriminations about the risk factors of poor prognosis between GNEC and GNET. For example, grade was the most important predictor for GNEC and it accounted for more points than any other factor in the nomogram, but it was not an independent prognostic factor of GNET. Previous study also indicated that grade was efficient to stratify type III GNENs, but was not associated with the prognosis of type I GNENs [16, 18]. Thus, we generated two separate nomograms to predict the survival of patients with different pathologic types and conducted a multicenter external validation with 246 GNENs patients from eight tertiary hospitals in Jiangsu province.

Total points calculated according to the established nomograms were served as new factor to predict the survival rates of GNEC and GNET patients. Excitingly, we achieved both excellent discrimination and calibration of the nomograms in our validation sets. The C-index of the nomogram was 0.743 for GNEC cohort and 0.945 for GNET cohort in Jiangsu. As for the AUC of the nomogram to predict the 3-year survival, it was up to 0.847 for GNEC and 0.960 for GNET, which were all better than those of AJCC staging system. Moreover, the calibration plots further confirmed the veracity between the nomogram-predicted survival rates and actual observed survival rates of Jiangsu GNENs patients, indicating the nomograms based on the SEER database were also suitable to patients in Jiangsu. However, 5-year OS of GNENs in Jiangsu was not validated for the reason that there was limited number of patients with a follow-up time up to 5 years. What’s more, the relatively short follow-up time of GNEC may also be attributed to its poor prognosis. ENETS consensus indicated that survival of gastrointestinal NEC ranged from 38 months for patients with localized disease to 5 months in the metastatic setting and only 5% of all patients were long-time survivors [18].

There are some limitations of our study. Firstly, RINDI classification of GNENs was unavailable in SEER database, impeding the opportunity to further research on prognosis of patients with different clinical types. In addition, the median follow-up time of Jiangsu GNENs patients was relatively short and sample size was relatively small, making it impossible to validate the 5-year OS in Jiangsu GNENs patients. Moreover, GNENs classification in SEER database was not completely equivalent to WHO classification. Finally, medical treatments for GNENs, such as chemotherapy and somatostatin analogues, were also not included.

In conclusion, the incidence of the GNENs is increasing steadily in the past 4 decades, especially in the localized and grade I tumors. We established two nomograms to predict the overall survival of GNEC and GNET separately. The result may provide valuable predicted message in clinical practice.

## Electronic supplementary material

Below is the link to the electronic supplementary material.

Supplementary material 1 (DOCX 11 kb)

Supplementary material 2 (PDF 684 kb)

Supplementary material 3 (PDF 156 kb)
