# Supplementary material for: Trends of incidence and prognosis of gastric neuroendocrine neoplasms: a study based on SEER and our multicenter research
Source: Gastric Cancer. 2020 Feb 5;23(4):591–9. doi: 10.1007/s10120-020-01046-8 (PMC7305263; doi:10.1007/s10120-020-01046-8)
Supplement: Supplementary file 1 — Supplementary material 1 (DOCX 11 kb) [file 10120_2020_1046_MOESM1_ESM.docx]

Supplementary Figure 1: Changes in the incidence of GNENs. The age-adjusted incidence of GNENs has increased steadily from 0.309 /1,000,000 in 1975 to 6.149/1,000,000 in 2016 with an AAPC of 7.2 %. In the period from 1975-2002, the APC was significantly different from that in period of 2002-2016(8.6% vs 4.7%, P<0.05).

Supplementary Figure 2: Kaplan-Meier survival curves of GNEC patients in SEER database.

Survival curves of GNEC by gender(A), age(B), grade(C), size(D), tumor site(E), T staging(F), N staging(G), M staging(H), AJCC staging(I), surgical methods(J).

Supplementary Figure 3: Kaplan-Meier survival curves of GNET patients in SEER database.

Survival curves of GNET by gender(A), age(B), grade(C), size(D), tumor site(E), T staging(F), N staging(G), M staging(H), AJCC staging(I), surgical methods(J).
