# Supplementary material for: Trends of incidence and prognosis of gastric neuroendocrine neoplasms: a study based on SEER and our multicenter research
Source: Gastric Cancer. 2020 Feb 5;23(4):591–9. doi: 10.1007/s10120-020-01046-8 (PMC7305263; doi:10.1007/s10120-020-01046-8)
Supplement: Supplementary file 2 — Supplementary material 2 (PDF 684 kb) [file 10120_2020_1046_MOESM2_ESM.pdf]

Article title:

Trends of incidence and prognosis of gastric neuroendocrine neoplasms-a study based on SEER and our multicenter research

Journal name: Gastric cancer

Author names and affiliations:

Ping Hu[1], Jian′ an Bai[1], Min Liu[1], Jingwen Xue[1], Tiaotiao Chen[1], Rui Li[2], Xiaoling Kuai[3], Haijian Zhao[4], Xiaolin Li[1], Ye Tian[1], Wei Sun[5], Yujia Xiong[2], Qiyun Tang[1]

1The First Affiliated Hospital of Nanjing Medical University

2Rui Li: The First Affiliated Hospital of Suzhou University

3Xiaoling Kuai: Affiliated Hospital of Nantong University

4Haijian Zhao: The Second People′ s Hospital of Huai′ an

5Wei Sun: Huai′ an First People′ s Hospital

2Yujia Xiong: The First Affiliated Hospital of Suzhou University

E-mail address of the corresponding author: tqy831@163.com

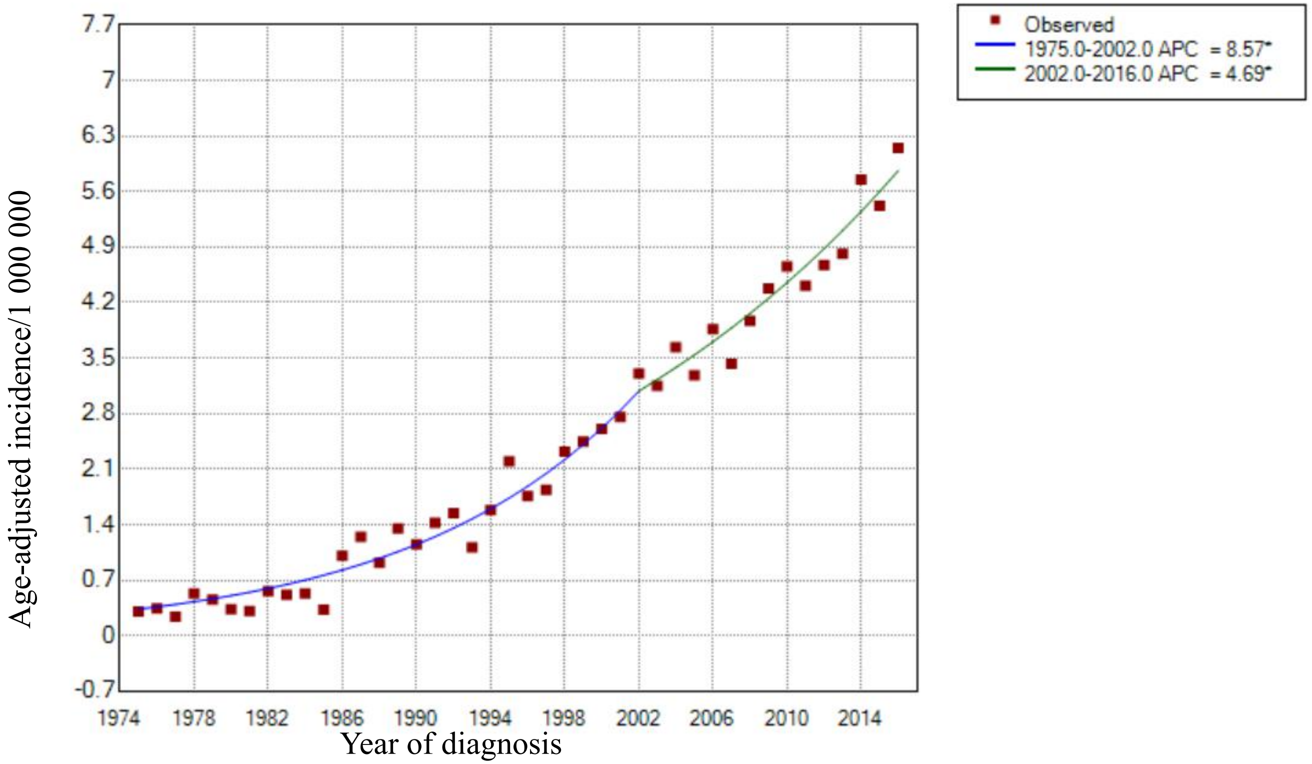

*Indicates that the Annual Percent Change(APC) is significantly different from zero at the alpha=0.05 level.

Final Selected Model: 1 Jionpoint

Supplementary Figure 1

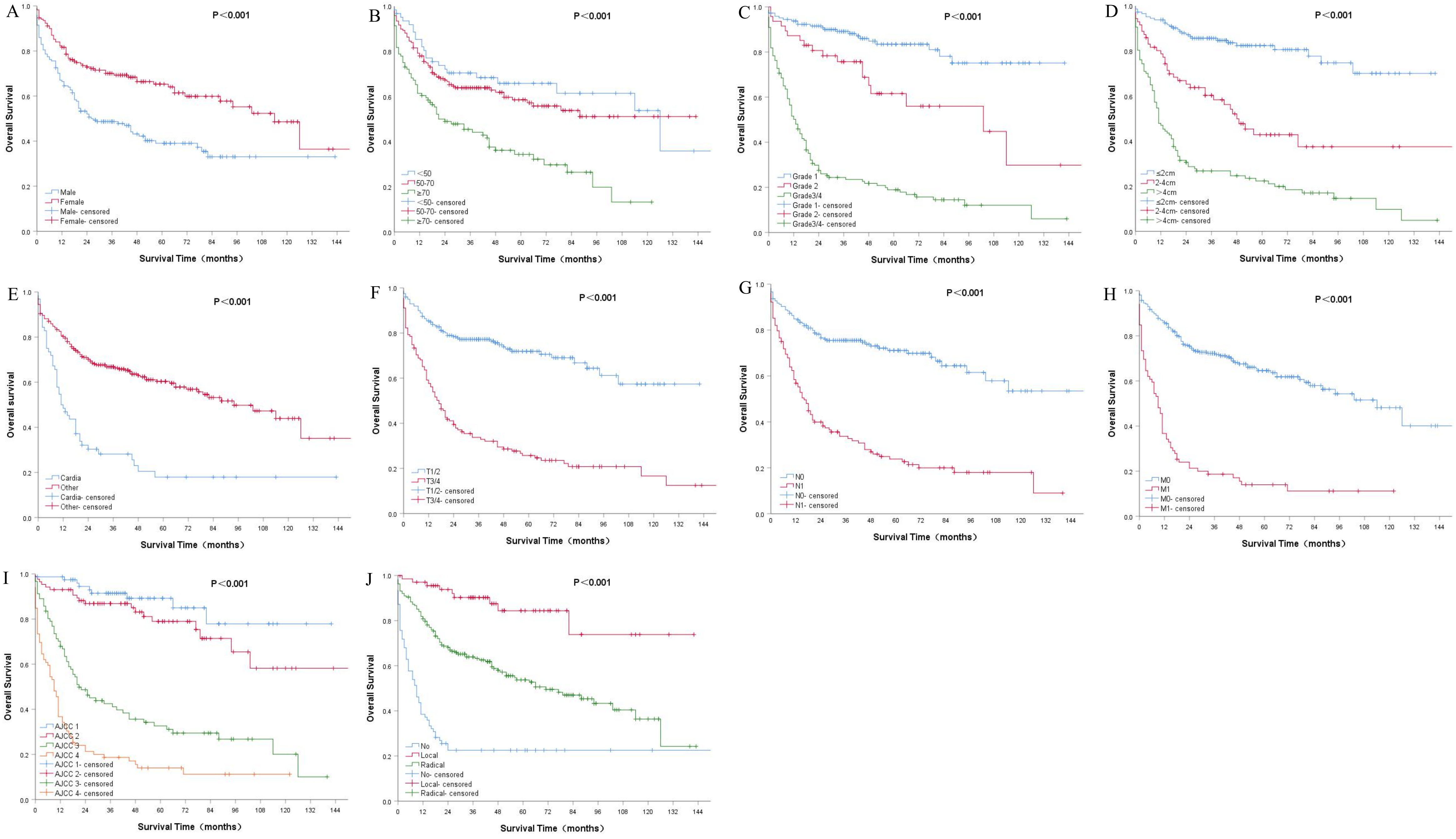

Supplementary Figure 2

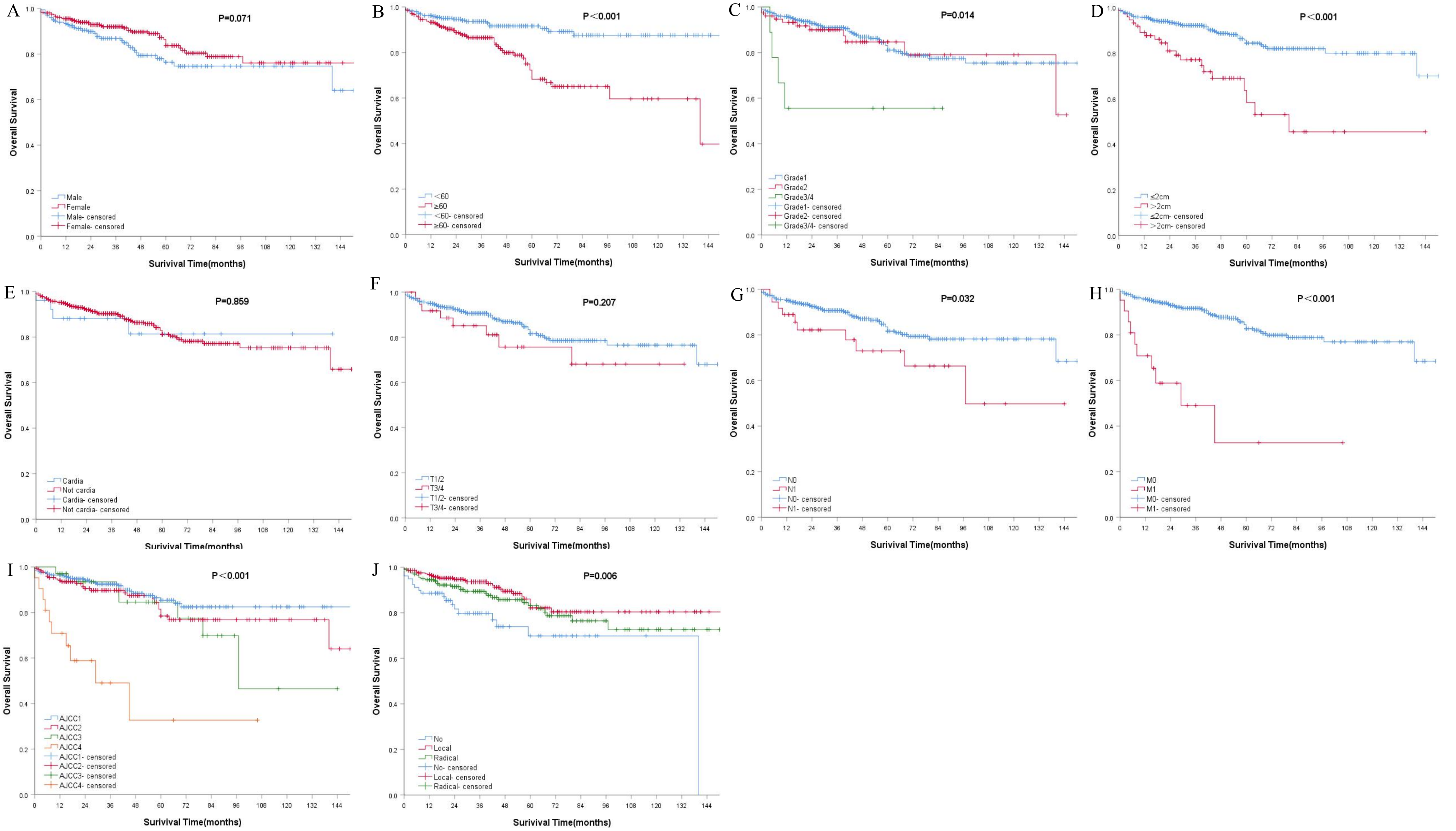

Supplementary Figure 3
