# Supplementary material for: Trends of incidence and prognosis of gastric neuroendocrine neoplasms: a study based on SEER and our multicenter research
Source: Gastric Cancer. 2020 Feb 5;23(4):591–9. doi: 10.1007/s10120-020-01046-8 (PMC7305263; doi:10.1007/s10120-020-01046-8)
Supplement: Supplementary file 3 — Supplementary material 3 (PDF 156 kb) [file 10120_2020_1046_MOESM3_ESM.pdf]

Article title: Trends of incidence and prognosis of gastric neuroendocrine neoplasms-a study based on SEER and our multicenter research

Journal name: Gastric cancer

Author names and affiliations:

Ping Hu[1], Jian’an Bai1, Min Liu[1], Jingwen Xue[1], Tiaotiao Chen[1], Rui Li[2], Xiaoling Kuai[3], Haijian Zhao[4], Xiaolin Li[1], Ye Tian[1], Wei Sun[5], Yujia Xiong[2], Qiyun Tang[1]

1The First Affiliated Hospital of Nanjing Medical University

2Rui Li: The First Affiliated Hospital of Suzhou University

3Xiaoling Kuai: Affiliated Hospital of Nantong University

4Haijian Zhao: The Second People’s Hospital of Huai’an

5Wei Sun: Huai’an First People’s Hospital

2Yujia Xiong: The First Affiliated Hospital of Suzhou University

E-mail address of the corresponding author: tqy831@163.com

Supplementary Table： Demographic or clinical characteristics of patients with GNENs in both training and validation sets

	GNEC				GNET			
	Training Set (n=334)		Validation Set (n=139)		Training Set (n=566)		Validation Set (n=107)	
	Cases	Proportion （%）	Cases	Proportion （%）	Cases	Proportion （%）	Cases	Proportion （%）
**Median Follow up time**		30.5(0-153)		27.0(2-89)		36.0(1-152)		40.0(6-107)
**Median OS**		71		41		＞150		＞107
**Gender**								
Male	164	49.10	117	84.17	202	35.69	49	45.79
Female	170	50.90	22	15.83	364	64.31	58	54.21
**Age**								
Median		63(21-89)		66(42-82)		59(17-85)		55(17-89)
Mean		61.93±13.52		65.17±8.95		59.97±12.78		55.57±1.33
＜50	62	18.56	8	5.76	294*	51.94*	65*	60.75*
50-69	168	50.30	83	59.71				
≥70	104	31.14	48	34.53	272^	48.06^	42^	39.25^
**Race**								
Black	43	12.87	0	0	80	14.13	0	0
White	260	77.84	0	0	460	81.27	0	0
Other	31	9.28	139	100.00	26	4.59	107	100

**Site**								
Cardia	64	19.16	68	48.92	26	4.59	15	14.02
Not Cardia	270	80.84	71	51.08	540	95.41	92	85.98
**Size**								
≤2cm	146	43.71	10	7.19	492	86.93	85	79.44
2-4cm	71	21.26	82	58.99				
＞4cm	117	35.03	47	33.81	74#	13.07#	22#	20.56#
**Grade**								
G1	144	43.11	0	0	481	84.98	71	66.36
G2	47	14.07	0	0	76	13.43	26	24.30
G3/4	143	42.81	139	100.00	9	1.59	10	9.35
**T staging**								
T1/2	199	59.58	24	17.27	529	93.46	95	88.79
T3/4	135	40.42	115	82.73	37	6.54	12	11.21
**N staging**								
N0	206	61.68	40	28.78	530	93.64	95	88.79
N1	128	38.32	99	71.22	36	6.36	12	11.21
**M staging**								
M0	255	76.35	92	66.19	545	96.29	99	92.52
M1	79	23.65	47	33.81	21	3.71	8	7.48
**AJCC staging**								
I	78	23.35	4	2.88	337	59.54	58	54.21

II	86	25.75	20	14.39	174	30.74	33	30.84
III	91	27.25	68	48.92	34	6.01	8	7.48
IV	79	23.65	47	33.81	21	3.71	8	7.48
**Surgery**								
No surgery	78	23.35	6	4.32	79	13.96	5	4.67
Local	68	20.36	0	0	287	50.71	62	57.94
Radical	188	56.29	133	95.68	200	35.33	40	37.38

Notes: Considering different biological characteristics of GNEC and GNET, there were a little differences about grouping to minimize bias: *<60 years old; ^≥60 years old; #＞2cm

Abbreviation: GNENs: gastric neuroendocrine neoplasms; GNEC: gastric neuroendocrine carcinoma; GNET: gastric neuroendocrine tumor; OS: overall survival
